# Clinical Effectiveness of Home‐Based Telerehabilitation Program for Geriatric Hip Fracture Following Total Hip Replacement

**DOI:** 10.1111/os.13521

**Published:** 2022-11-29

**Authors:** Wei‐yong Wu, Yin‐guang Zhang, Yuan‐Yuan Zhang, Bing Peng, Wei‐guo Xu

**Affiliations:** ^1^ Department of Orthopaedics Tianjin Hospital Tianjin China; ^2^ Department of Hip Trauma Tianjin Hospital Tianjin China; ^3^ Department of Rehabilitation Tianjin Hospital Tianjin China

**Keywords:** Elderly patients, Hip fracture, Postoperative management, Telerehabilitation, Total hip replacement

## Abstract

**Objective:**

To compare the effectiveness of a six‐month home‐based telerehabilitation based on the Internet‐based rehabilitation management system coupled with conventional outpatient care in elderly patients with hip fractures following total hip replacement (THR).

**Methods:**

Elderly patients (aged over 65 years) with first hip fractures who underwent THR between March 2018 and September 2018 in Tianjin Hospital were enrolled in this study. Patients were divided into two groups: telerehabilitation group (n = 43) and telephone group (n = 42). A Internet‐based telerehabilitation management system was established and applied on patients in the telerehabilitation group. For patients in the telephone group, the rehabilitation intervention was administered through conventional outpatient care (telephone along with outpatient follow‐up). Data from the Harris hip scale (HHS), functional independence measure (FIM), self‐rating anxiety scale (SAS), and postoperative complications at 1, 3, and 6 months after surgery were collected and compared between the two groups.

**Results:**

A total of 85 elderly patients completed the 6‐month follow‐up assessment. Results showed that the HHS score was significantly higher in the telerehabilitation group than in the telephone group at 1 month (66.35 ± 4.63 *vs* 63.48 ± 4.49), 3 months (76.33 ± 4.52 *vs* 71.81 ± 3.84), and 6 months (84.23 ± 3.13 *vs* 77.29 ± 4.95) after surgery (*P* < 0.001). The FIM score was significantly higher in the telerehabilitation group than in the telephone group at 1 month (89.00 ± 5.63 *vs* 73.35 ± 8.70), 3 months (100.16 ± 4.56 *vs* 92.81 ± 5.17), and 6 months (111.70 ± 3.13 *vs* 98.64 ± 5.12) after surgery (*P* < 0.001). The SAS score was significantly lower in the telerehabilitation group than in the telephone group at 1 month (42.40 ± 3.07 *vs* 46.21 ± 3.53), 3 months (36.77 ± 2.26 *vs* 40.24 ± 1.66), and 6 months (29.26 ± 1.63 *vs* 33.81 ± 2.62) after surgery (*P* < 0.001). The overall complication rate was significantly lower in the telerehabilitation group than in the telephone group (14% *vs* 40.5%) (*P* < 0.05).

**Conclusion:**

Internet‐based rehabilitation management system can not only promote the physical rehabilitation of patients, but also play a positive role in psychological rehabilitation and the prevention of complications, which provides new ideas and methods for clinical rehabilitation.

## Graphical abstract

A total of 103 potentially eligible older patients (aged over 65 years) treated at Tianjin Hospital after total hip replacement (THR) was identified, of which 85 participants were enrolled in the study and were allocated into the telerehabilitation (n = 43) or telephone groups (n = 42). The reasons for exclusion and dropouts of participants during the data collection and per‐protocol analysis are provided in the flow diagram (Fig. 1).

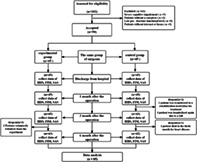

## Introduction

The World Health Organization defines “geriatric hip fracture” as a hip fracture in adults over 65 years of age caused by low‐energy injury. It has been reported that the mortality rate of elderly patients with hip fractures ranges between 14% and 36% within 1 year after onset. About 8%–28% of patients with hip fractures will develop one or more postoperative complications.^1^ The occurrence of postoperative complications may triple the mortality rate within 1 year and increase the difficulty of treating the disease.^2^, ^3^ Data show that total hip replacement (THR) reduces the length of time fractured hip patients stay in bed and the risk of post‐operative complications. The increase in the number of elderly patients has resulted in the rise in THR procedures worldwide.^4^, ^5^ Although THR reduces deaths due to hip fractures in elderly patients to some extent, 22–75% of patients with hip fracture undergoing THR do not regain their pre‐injury health status. Their physical condition remains poor, creating a huge socio‐economic burden. Also, such patients are immobilized for a long period after surgery, which exacerbates their underlying diseases and increases the risk of complications. Therefore, early rehabilitation intervention is essential to promote post‐operative recovery. Patients often experience poor prognosis after THR due to the lack of appropriate post‐operative rehabilitation.^6^

A good rehabilitation program should be gradual, Individualized, and comprehensive.^7^ Some scholars^8^ believe that the best time of rehabilitation after THR is from the first day until 6 months after the operation. The average length of hospital stay for elderly patients after THR is 2 weeks, and most of the rehabilitation is performed outside the hospital. In China, rehabilitation is mainly provided through telephone and outpatient visits. However, some rehabilitation methods cannot be conveyed accurately through telephone. For rehabilitation treatments given through outpatient visits, some patients abandon the treatment halfway due to the inconvenience of frequent movements. Therefore, scientists in China and other countries are investigating strategies to improve delivery of rehabilitation programs to elderly patients outside of the hospital set up.

Telemedicine is a method of providing health care services to patients from a distance using communication technologies.^9^ Telerehabilitation (TR) is a branch of telemedicine. TR is an emerging method meant to improve the self‐contained motor, cognitive, or psychological disabilities of the patient or family member at home.^10^ TR is even more advantageous to people living in remote areas or who cannot reach the care centers due to physical impairments.^11^ In addition, TR can be performed at any self‐determined time, therefore, adherence and compliance are improved. For elderly patients, TR seems to be an ideal approach since they have difficult visiting hospitals given their poor physical status. Therefore, TR seems to be particularly suitable for elderly patients with hip fracture after undergoing THR. Currently, TR has been mostly applied to treat medical conditions such as cardiac and pulmonary diseases.^12^, ^13^ Few studies have compared differences in THR outcomes between conventional outpatient care and TR practices for elderly patients.

Our research group has developed the family‐oriented postoperative rehabilitation management system for geriatric hip fracture (referred to as the “rehabilitation management system”). In our previous study^14^ we had proved that this system can enhance the ability to perform activities of daily living and somatic integration in the postoperative rehabilitation of hip fracture patients. However, in the operation of hip fracture, total hip arthroplasty has longer operation time and more blood loss, which is easy to cause postoperative complications.^15^ And there is a high risk of joint dislocation after THR.^16^ Because of the fear of joint dislocation, patients are easy to cause common obstacles to psychological function and limb function. Therefore, we conducted a study on patients with total hip replacement. This experiment not only focuses on the rehabilitation of limb function but also on the psychological function and postoperative complications of patients. On the basis of our previous study,^14^ we increased the follow‐up time to reflect the rehabilitation effect for a longer time. The following were the aims of this: (i) to investigate the therapeutic effect of home‐based telerehabilitation on postoperative hip function and functional independence; (ii) to determine whether home‐based telerehabilitation can effectively relieve psychological anxiety; and (iii) to analyze the effect of rehabilitation management system on preventing postoperative complications.

## Materials and Methods

### Inclusion and Exclusion Criteria

The participants were hip fracture patients hospitalized in the Tianjin hospital from January March 2018 to September 2018. This study was approved by the Ethics Committee of the Tianjin Hospital (2022–079).

Inclusion criteria: (i) elderly patients (aged over 65 years) with first hip fractures after THR; (ii) rehabilitation intervention was provided using the TR system in the telerehabilitation group; (iii) at least one person between the patient and the caregiver can skillfully operate the TR system; and (iv) Patients without mental disorders who can cooperate with rehabilitation treatment.

Exclusion criteria: (i) patients with severe cognitive impairment; (ii) patients with low pre‐fracture functional level before fracture; (iii) patients without a caregiver at home; and (iv) patients without internet at home.

Drop‐out criteria: (i) patients voluntarily withdrew from the experiment; (ii) patients treated by rehabilitation institutions during follow‐up; and (iii) patients who died during follow‐up.

### Participants

**Fig. 1:**
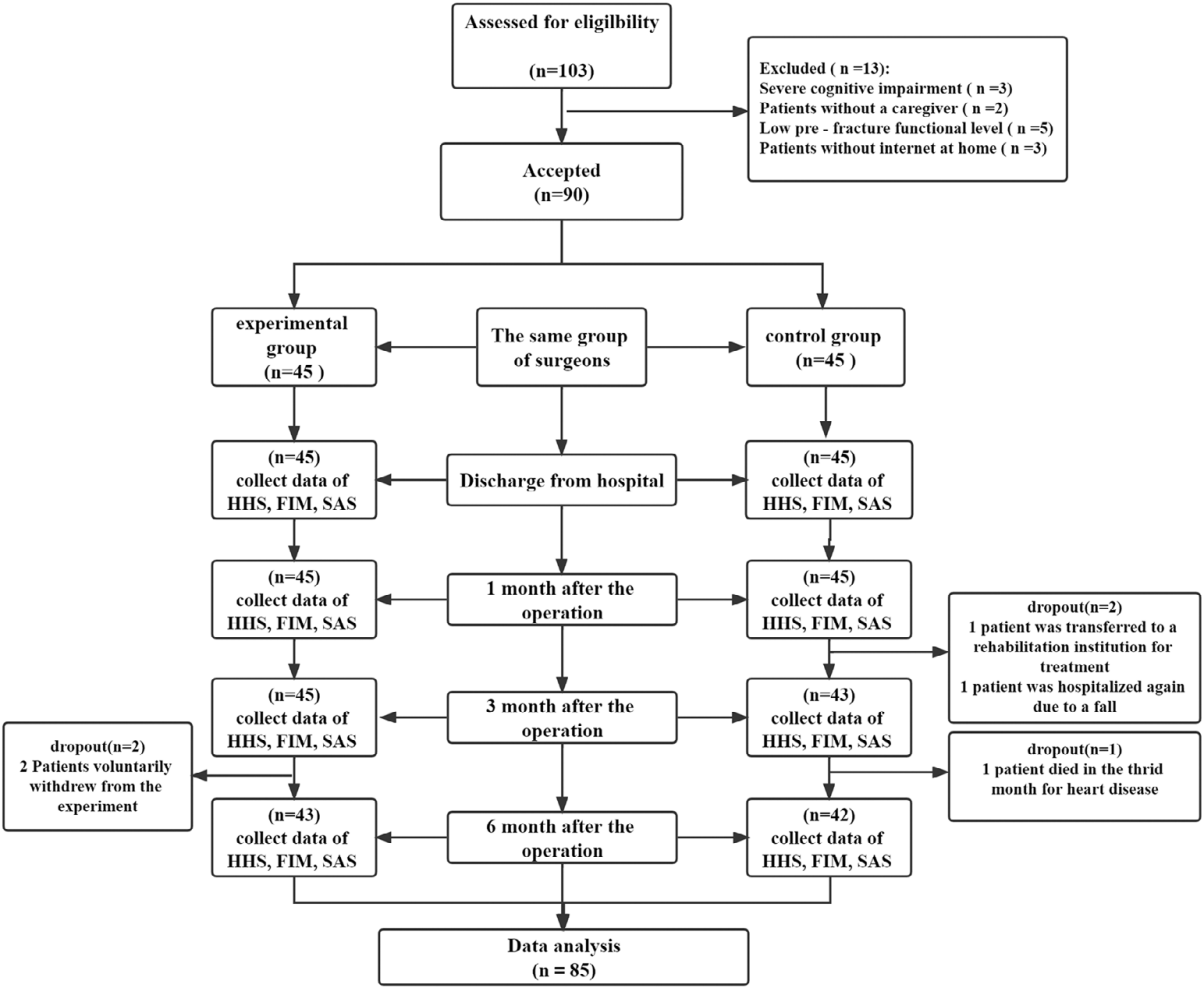
Flow chart for the inclusion process of the consolidated standard

A total of 103 potentially eligible older patients (aged over 65 years) treated at Tianjin Hospital after THR were identified, of which 85 participants were enrolled in the study and were allocated into the telerehabilitation (n = 43) or telephone groups (n = 42). The reasons for exclusion and dropouts of participants during the data collection and per‐protocol analysis are provided in a flow diagram (Fig. 1).

### Interventions

**Fig. 2:**
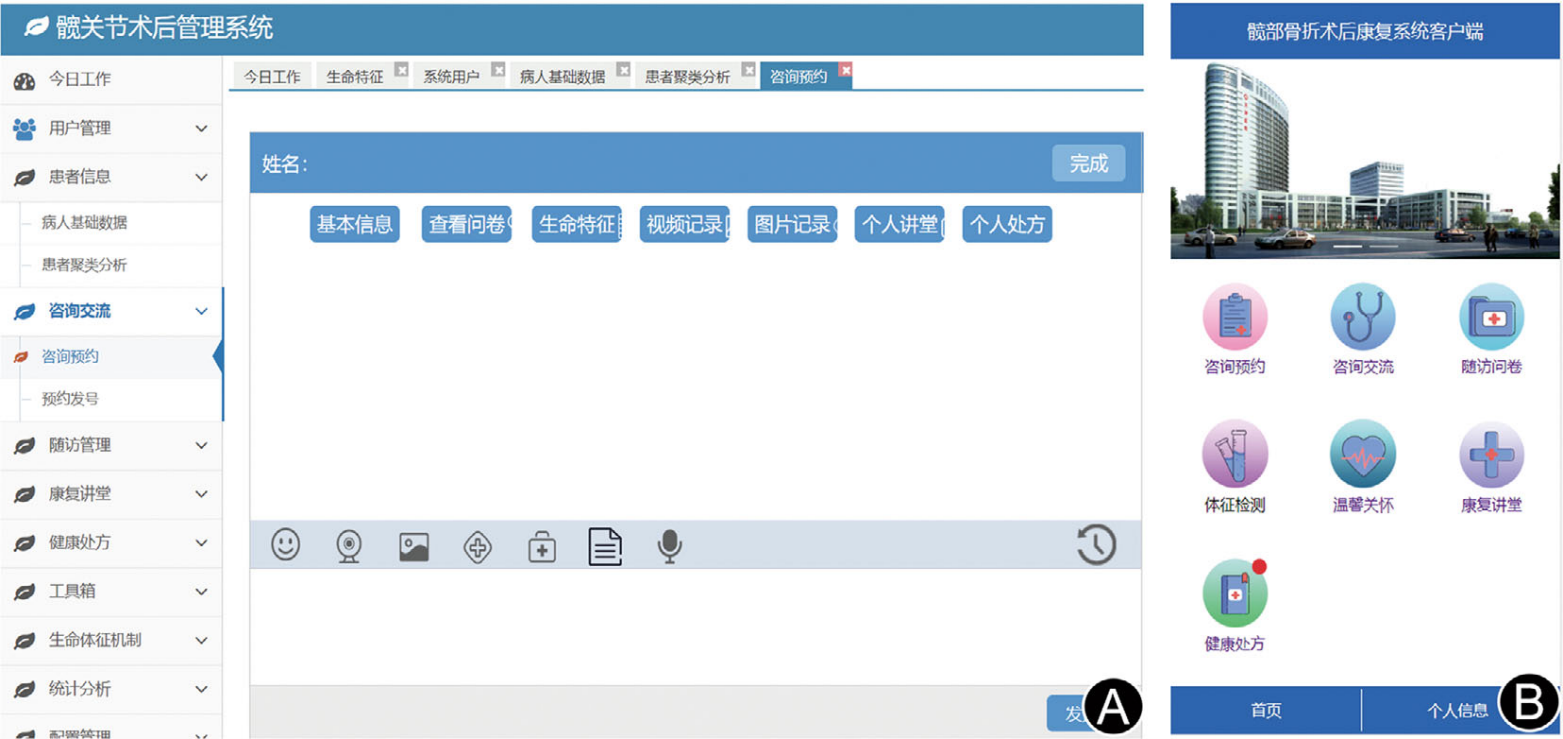
The hip fracture post‐operative management system; (A): system operation interface for the doctor; (B): system operation interface for patient

**Fig. 3:**
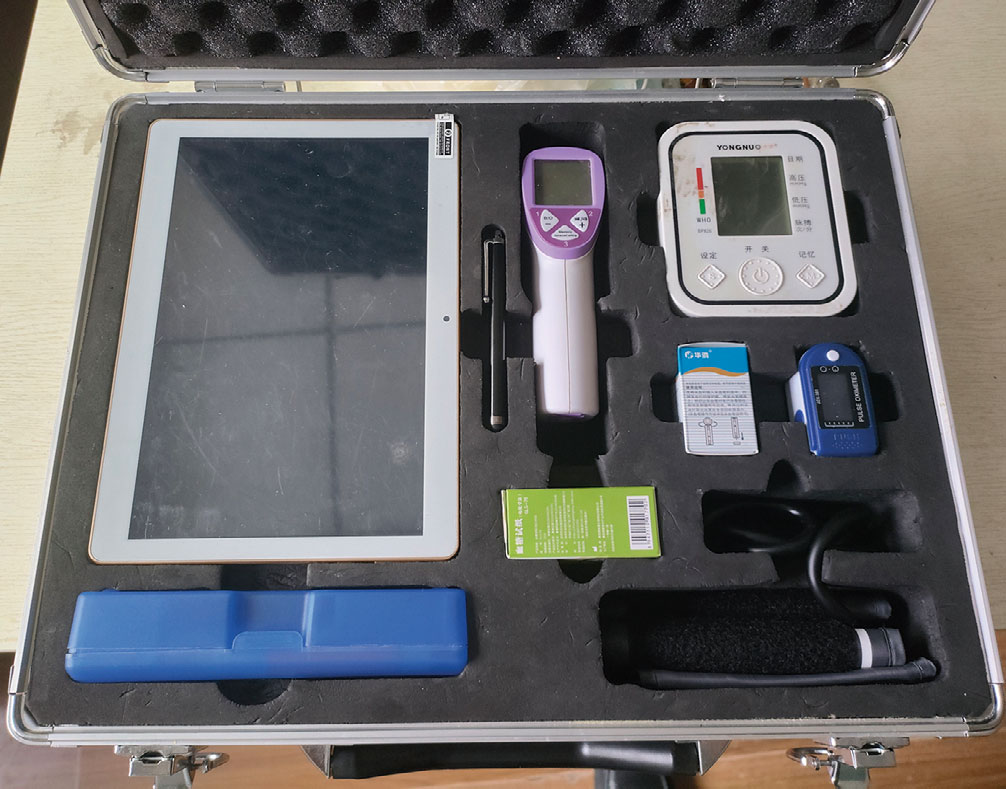
The rehab box including a microcomputer and Bluetooth‐connectable peripherals

The “Family‐oriented postoperative rehabilitation management system for geriatric hip fracture” (referred to as “rehabilitation management system”) is similar to a mini‐type hospital information system. Through this system, discharged patients can be managed uniformly, and the medical services can be extended to the patient's home. This system allows doctors to issue health prescriptions to patients, mainly, by providing general information about orthopedic rehabilitation treatment, precautions for rehabilitation treatment, rehabilitation actions at different rehabilitation stages, etc. Relevant information services can be provided by text, pictures, videos, and other means. The system provides video communication technology. Through video communication, medical personnel can effectively understand the rehabilitation status of patients. The system has multiple types of terminals consisting of an application (APP) for patients and a working portal for the doctor in the clinic (Fig. 2). The main component of the system from the patient's terminal is the rehab box, which includes a microcomputer with internet access and bluetooth‐connectable peripherals (electronic thermometer, electronic blood pressure meter, electronic blood glucose meter, and electronic blood oxygen meter; Fig. 3). The application is installed on the minicomputer in the rehab box. The doctor's terminal is the personal computer, which is connected to the system through the corresponding website.

#### Telerehabilitation Group

All patients in the telerehabilitation group were assigned to “the hip fracture post‐operative management system.” After the patients in the telerehabilitation group were discharged, they established contact with the hospital through this system. Patients also returned to the hospital for regular X‐ray examination at 1, 3, and 6 months after the operation. The intervention contents are as follows.

Multidisciplinary interventions: the multidisciplinary group consists of a physical therapist, orthopedic surgeons, nurses, and geriatricians. The physical therapist formulates the rehabilitation plan. Orthopedic surgeons make minor adjustments according to the patient's operation mode. The geriatrician focuses on prevention and appropriate interventions for internal diseases. The nurse is responsible for implementing and supervising the rehabilitation plan.

Medical information pushing: a video of the rehabilitation actions expressed initially in words in the post‐operation rehabilitation program is prepared according to previous literature^17^ (Table 1). Based on different rehabilitation stages, rehabilitation videos that patients can tolerate (including the safest way to perform activities of daily living, a description of self‐care activities and walking aids, cardiopulmonary function exercise, balance exercises, etc.) are regularly sent. Patients watch the video repeatedly until they master it.

**TABLE 1:** Post‐op rehabilitation program

1–2 weeks after the operation	1. Ankle Exercises (Move your ankles up and down and around in circles.) 2. Isometric Quadriceps Contractions (Tighten the muscle in the front of your thigh by pushing the back of your knee into the bed and pulling your toes towards you.) 3. Hip and Knee Bending (Bend your operated hip and your knee keeping your foot on the bed but do not bend your hip past 90°.) 4. Hip Abduction (Keep your knee straight, slide your operated leg out to the side but do not move your leg past the midline.)
3–7 weeks after the operation	1. Bridging (Bend both your knees and push your feet into the bed and raise your bottom off the bed.) 2. Hip Extension (Hold onto a towel rack, kitchen bench, or solid piece of furniture. Stand erect and keep the knee straight, move the operated leg out to the back.) 3. Hip Abduction (Stand with feet slightly apart. Keep knee straight move operated leg out to the side.) 4. Standing toe raises (While standing on the floor and holding the grab bars with your hands, roll up onto the balls of your feet, lifting your heels off the ground and keeping your knees and your back straight, and then roll down slowly.)
8–12 weeks after the operation	1. Hip muscle exercises (Hold on to the grab bar with your left hand. Raise your right leg to the side 45°and hold it, and then lower it slowly.) 2. Leg lifts (Place a 1–2 kg weight on the ankle. Lie flat on the bed and lift your toes to the ceiling so your knee is straight.) 3. Stair dips (Hold onto the grab bars, lower your operated leg down to the floor while your other leg is still on the top step above it, and then bring your foot back up to the step.) 4. daily activities Encourage patients to perform daily functional activities
After 12 weeks	1. Guidance of traditional Chinese medicine: Such as Tai Chi, Wuqinxi, and Baduanjin. 2. Do some simple housework and outdoor activities.

*Note*: You must exercise at least 3 times daily. Repeat each exercise slowly at least 5–10 times.

Data reporting: patients can send text messages and doctors can reply at their convenience. Patients or their families record videos of patients' recovery progress for the corresponding rehabilitation stage twice a week and upload the video to the system. They use the Bluetooth‐connectable peripherals for daily check‐ups by themselves. The measured data are uploaded to the system. The rehabilitation team can log in to the system to view these data at any time.

Consultation and communication: patients and doctors should conduct video conferences weekly. Patients can ask questions about their training. Regarding patients' psychology, the doctor communicating with the patient should focus on alleviating patients' anxiety and encourage patients to complete training tasks as planned. The doctor should also understand the occurrence of complications (wound infection, bedsore, joint dislocation, etc.) through video conferences and provide corresponding guidance and preventive measures.

#### Telephone Group

Conventional outpatient care: upon discharge, patients with hip fractures are provided with a text version of the post‐operative rehabilitation program (Table 1) and are taught all‐action standards by physical therapists. Patients are followed up weekly by telephone and their questions answered. The patients return to the clinic for X‐ray examination and assessment of recovery status at 1, 3, and 6 months after the operation. The therapist instructs the patient in the outpatient department on the concrete specific rehabilitation methods and exercises criteria for the corresponding rehabilitation stage.

### Evaluation Criteria

All patients and their caregivers were investigated for functional parameters at Tianjin Hospital and were assessed at four‐time points: (i) discharge from hospital; (ii) 1 month after the operation; (iii) 3 months after the operation; and (iv) 6 months after the operation (end of the TR program). All patients reported postoperative complications (including adverse events) weekly during the experiment (the telerehabilitation group *via* the rehabilitation management system and the telephone group *via* telephone). Follow‐up data were collected by a full‐time sports scientist who was blinded to group allocation. Data analysis was performed by a statistician, who was blinded to group allocation. The physical function of patients was assessed using the Harris hip scale (HHS)^18^ and functional independence measure (FIM).^19^ The self‐rating anxiety scale (SAS)^20^ was used to evaluate psychological recovery. The overall incidence of postoperative complications within 6 months was calculated and used to assess the comprehensive recovery.

### Statistical Analysis

All data were analyzed using SPSS 22.0 software (IBM, Armonk, NY, USA). Continuous data with normal distribution were expressed as mean ± standard deviation (mean ± SD). An independent‐samples *t*‐test was used for intergroup comparison. Categorical variables in both groups were analyzed using the chi‐square test. Repeated‐measures analysis of variance (ANOVA) was used to analyze the longitudinal change trend of each index at different time points after discharge. For all tests, *P* values <0.05 were considered statistically significant.

## Result

### General Results

The general demographic information of included patients are presented in Table 2. A total of 85 participants (43 in the telerehabilitation group and 42 in the telephone group) were included in the final data analysis. The average age of patients was 74.28 ± 5.06 years (13 [30.2%] men and 30 [69.8%] women) in the telerehabilitation group and 72.00 ± 6.77 years (11 [26.2%] men and 31 [73.8%] women) in the telephone group. There was no statistical difference between the two groups in age (*P* = 0.579), gender (*P* = 0.679), fracture classification (*P* = 0.669), Prosthesis type (*P* = 0.305), affected side (*P* = 0.915), types of co‐morbidity (*P* = 0.857) and ASA rating (*P* = 0.890).

**TABLE 2:** Characteristics of participants

Variables	Telerehabilitation group (n = 43)	Control group (n = 42)	t/*χ* ^2^	*P* value
Age (years)	74.28 ± 5.06	72.00 ± 6.77	0.557	0.579
Gender, (n)			0.171	0.679
Men	13	11		
Women	30	31		
Fracture classification, (n)			0.182	0.669
The neck of the femur	40	38		
Intertrochanteric fracture	3	4		
Prosthesis type, (n)			1.054	0.305
Bone cement	19	14		
Non‐bone cement	24	28		
Affected side, (n)			0.011	0.915
Left leg	22	21		
Right leg	21	21		
Types of co‐morbidity, (n)			1.182	0.857
No	4	13		
One type	11	10		
Two type	23	22		
Three or more	5	7		
ASA rating			0.234	0.890
I	7	8		
II	20	21		
III	15	13		

Note: Control group, rehabilitation was provided through conventional outpatient care; Telerehabilitation group, rehabilitation was provided using the rehabilitation management system.

### Comparison of HHS Scores

No significant difference in HHS scores was found between the telerehabilitation and telephone groups at discharge (*P* > 0.05). HHS scores increased gradually in the two groups over time. However, the HHS score was significantly higher in the telerehabilitation group than in the telephone group at 1 month (66.35 ± 4.63 *vs* 63.48 ± 4.49), 3 months (76.33 ± 4.52 *vs* 71.81 ± 3.84), and 6 months (84.23 ± 3.13 *vs* 77.29 ± 4.95) after the operation (*P* < 0.001, Table 3).

**TABLE 3:** Comparison of HHS scores between the two groups

Groups	Discharge from hospital	1 month after the operation	3 month after the operation	6 month after the operation
Telerehabilitation group	43.60 ± 6.09	66.35 ± 4.63	76.33 ± 4.52	84.23 ± 3.13
Control group	42.50 ± 5.27	63.48 ± 4.49	71.81 ± 3.84	77.29 ± 4.95
t value	0.893	2.900	4.957	7.757
*p*‐value	0.374	0.005	0.00	0.00

*Note*: Time‐dependent effect: F = 1324.609, *P* < 0.01; Group‐dependent effect: F = 35.130, *P* < 0.01; Interaction effect: F = 7.571, *P* < 0.01.

### Comparison of FIM Scores

No significant difference in FIM scores was found between the telerehabilitation and telephone groups at discharge (*P* > 0.05). FIM scores increased gradually in the two groups over time. However, the FIM score was significantly higher in the telerehabilitation group than that in the telephone group at 1 month (89.00 ± 5.63 *vs* 73.35 ± 8.70), 3 months (100.16 ± 4.56 *vs* 92.81 ± 5.17), and 6 months (111.70 ± 3.13 *vs* 98.64 ± 5.12) after the operation (*P* < 0.001, Table 4).

**TABLE 4:** Comparison of FIM scores between the two groups

Groups	Discharge from hospital	1 month after the operation	3 month after the operation	6 month after the operation
Telerehabilitation group	62.47 ± 3.52	89.00 ± 5.63	100.16 ± 4.56	111.70 ± 3.13
Control group	62.36 ± 4.61	73.35 ± 8.70	92.81 ± 5.17	98.64 ± 5.12
t value	0.122	3.146	6.957	10.616
*p*‐value	0.904	0.005	0.00	0.00

Note: Time‐dependent effect: F = 1532.885, *P* < 0.01; Group‐dependent effect: F = 59.007, *P* < 0.01; Interaction effect: F = 33.388, *P* < 0.01.

### Comparison of SAS Scores

No significant difference in SAS scores was found between the telerehabilitation and telephone groups at discharge (*P* > 0.05). SAS scores decreased gradually in the two groups over time. However, the SAS score of the telerehabilitation group was significantly lower than that of the telephone group at 1 month (42.40 ± 3.07 *vs* 46.21 ± 3.53), 3 months (36.77 ± 2.26 *vs* 40.24 ± 1.66), and 6 months (29.26 ± 1.63 *vs* 33.81 ± 2.62) after the operation (*P* < 0.001, Table 5).

**TABLE 5:** Comparison of SAS scores between the two groups

Groups	Discharge from hospital	1 month after the operation	3 month after the operation	6 month after the operation
Telerehabilitation group	53.12 ± 4.72	42.40 ± 3.07	36.77 ± 2.26	29.26 ± 1.63
Control group	52.45 ± 4.94	46.21 ± 3.53	40.24 ± 1.66	33.81 ± 2.62
t value	0.633	5.324	8.032	9.626
*p*‐value	0.528	0.00	0.00	0.00

Note: Time‐dependent effect: F = 881.722, *P* < 0.01; Group‐dependent effect: F = 33.922, *P* < 0.01; Interaction effect: F = 15.010, *P* < 0.01.

### Comparison of Post‐Operative Complications

Within 6 months after discharge, 23 patients developed complications, including six patients (14%) in the telerehabilitation group and 17 patients (40.5%) in the telephone group (two patients in the telephone group had two complications). The incidence of complications was significantly lower in the telerehabilitation group compared with the telephone group (*P* < 0.05, Table 6).

**TABLE 6:** Comparison of complications between the two groups of patients

Characteristics	Telerehabilitation group (n = 43)		Control group (n = 42)		*χ* ^2^	*P* value
	Number(n)	Percentages(%)	Number(n)	Percentages(%)		
Wound infection	2	4.7%	6	14.3%	1.321	0.25
Dislocation	0	‐	1	2.3%	1.036	0.494
Thrombosis	2	4.7%	4	9.5%	0.206	0.65
Pneumonia	1	2.3%	3	7.1%	0.288	0.592
Urinary tract infection	1	2.3%	3	7.1%	0.288	0.592
Decubitus ulcer	0	‐	2	4.8%	2.097	0.241
Total complications	6	14%	17	40.5%	7.573	0.006

## Discussion

The enrolled patients were over 65 years old. About 71.7% were women. It has been previously reported that the prevalence of osteoporosis and related fractures is higher in postmenopausal women. The proportion of patients suffering from comorbidities before the operation was 78.8%, of which 35.2% had more than two types of comorbidities, which is generally consistent with the epidemiological survey results.^21^, ^22^ The physical function of elderly patients declines to varying degrees, which increases the risk of comorbidities and post‐operative disability. Therefore, it is crucial to actively provide formal rehabilitation intervention for elderly patients after the operation. The goal of rehabilitation treatment is to reduce the disability rate and improve patients' quality of life. However, many patients cannot access adequate and appropriate rehabilitation services after discharge due to poor mobility and transport challenges. On this basis, this study aimed to evaluate the benefits of telerehabilitation.

### Effect on Postoperative Hip Function and Functional Independence

HHS and FIM scores were higher in the telerehabilitation group than in the telephone group at each stage after discharge. The scores improved gradually with time (*P* < 0.001). This shows that compared with the outpatient rehabilitation approach, application of the hip fracture post‐operative management system has better effects in terms of improved hip function and restoring patient independence. Moreover, the information about the rehabilitation in the telerehabilitation group was collected through pictures and videos, which are more accurate compared to the asymmetric information obtained through oral review at the clinic. The TR breaks the boundaries of time and space which allows doctors monitor the rehabilitation status of patients anytime and anywhere. This also allows doctors to provide timely treatment according to the patient tolerance. Research^23^, ^24^, ^25^ has shown that supervised rehabilitation provides better therapeutic effects on recovery of hip joint function than unsupervised one in orthopedic rehabilitation.^26^ Patients in the telerehabilitation group performed their prescribed daily exercise plan in the company of family members. This increases compliance to the rehabilitation program. It also helps patients regain their self‐care ability and walking ability faster. In 2014, a study reported that the use of a home‐based exercise program resulted in modest improvement in physical function at 6 months after intervention.^27^ The World Federation of Occupational Therapists (WFOT)^11^ issued a position statement on the use of telemedicine to improve the possibility of returning patients to society. Telemedicine is considered to be an effective and reliable approach to restore independence among patients.

### Effect of Relieving Psychological Anxiety

Patients with THR may experience anxiety due to wound pain, fear of poor limb function recovery, fear of prosthesis dislocation, and other inconveniences associated with the disease. In our study, SAS scores of patients after hip surgery were higher, indicating that hip fractures increased anxiety levels among patients. However, the SAS score gradually decreased after rehabilitation. The SAS score of the telerehabilitation group was significantly lower than that of the telephone group at all time points (*P* < 0.001). This implied that TR gradually decreased the degree of anxiety among patients which is in agreement with findings from other studies.^28^, ^29^, ^30^ The home‐based TR program was developed to enable patients to complete rehabilitation training at home rather than at institutions. While undergoing rehabilitation at home, patients are exposed to an environment where they can engage in same living activities as before the illness. The familiar and comfortable home environment coupled with the support from family members makes it easier for patients to recover.^31^, ^32^ The home‐based telerehabilitation can reduce patients' fear of the hospital environment which accelerates their recovery compared to patients using drug intervention only. Consistent with the present results, previous studies have confirmed that patients who complete rehabilitation training at home have higher confidence levels, low anxiety, and hence better rehabilitation results.^33^ In addition, establishment of a suitable communication mechanism will help alleviate anxiety after the operation. Communicating with patients weekly increases their mental power to confront the disease by eliminating negative emotions about the disease and treatment. Moreover, the video communication incorporated in the system increases patients' trust and compliance, as well as alleviates patients' anxiety.

### Effects of Preventing Post‐operative Complications

At the last follow‐up (6 months after operation), the total incidence of complications in the telerehabilitation group was 14%, which was significantly lower than that in the telephone group (40.5%). Elderly patients have poor physical state (i.e., many illnesses and poor surgical tolerance). These conditions may aggravate the underlying conditions and increase the risk of complications after surgery.^34^ The home‐based TR program allows patients to receive multidisciplinary assistance at home. The therapists can formulate a rehabilitation plan according to the patient's rehabilitation progress, and the surgeon can make adjustments according to the patient's situation to achieve personalized rehabilitation. Geriatricians can access the patient's vital signs information through the system and are hence, able to understand the patient's physical condition and thereby provide effective treatment. Nurses are responsible for implementing and supervising the rehabilitation plan to ensure patient compliance. In a recent prospective cohort study, multidisciplinary treatments reduced the incidence of postoperative complications, prevented secondary fractures, and ultimately decreased mortality rates in elderly patients with hip fractures.^35^ The use of the rehabilitation management system to provide rehabilitation guidance is crucial to achieving effective self‐management of geriatric hip fractures. A recent study showed that telemedicine achieved self‐management of chronic diseases (rheumatic system diseases) and obtained high patient satisfaction.^36^ Telerehabilitation improved health management of basic diseases and allowed timely telephone of postoperative complications to avoid adverse consequences.

## Limitations

Due to telerehabilitation conditions and time constraints, the intervention time in this study is short, thus the long‐term effects of TR on patients with hip fracture after THR were not investigated. In addition, we were unable to blind participants and investigators. Patients and their caregivers were not blinded to treatment allocation, which may have influenced the results. Finally, this study was conducted in one hospital; therefore, multi‐center studies should be conducted in the future to validate the present findings.

### Conclusion

Internet‐based rehabilitation management system provides a new approach for implementing rehabilitation at home to elderly patients with hip fracture following THR. The home‐based TR program provide the following: (i) it allows patients to receive continuous rehabilitation treatment; without skipping (ii) multiple communication mechanisms (word + video) provided in the system makes it easier for patients to master the key points of rehabilitation; and (iii) the program allows early prevention and intervention of complications. In addition, the Internet‐based rehabilitation management system can be applied remotely to the participant's home making it universally available. Given that the technology and equipment (the APP installed on the minicomputer) are readily available and easy to use, it is possible to popularize the adoption of Internet‐based rehabilitation management system in clinical practice.

## Author Contributions

Wei‐guo Xu was major contributor to study design and critically reviewed for important intellectual content. Yin‐guang Zhang supervised and directed the experimental process. Wei‐yong Wu was involved in collecting data, wrote and revised the draft of the manuscript. Yuan‐yuan Zhang and Bing Peng assisted with data acquisition and analysis. Both authors approved the final manuscript and agree to be accountable for all aspects of the work.

## References

[1] de Palma L , Torcianti M , Meco L , Catalani A , Marinelli M . Operative delay and mortality in elderly patients with hip fracture: an observational study. Eur J Orthop Surg Traumatol. 2014;24:783–8. 23712671 10.1007/s00590-013-1241-y

[2] Shen JW , Zhang PX , An YZ , Jiang BG . Prognostic implications of preoperative pneumonia for geriatric patients undergoing hip fracture surgery or arthroplasty. Orthop Surg. 2020;12:1890–9. 33112045 10.1111/os.12830 PMC7767666

[3] Jiang Y , Luo Y , Lyu H , et al. Trends in comorbidities and postoperative complications of geriatric hip fracture patients from 2000 to 2019: results from a hip fracture cohort in a tertiary hospital. Orthop Surg. 2021;13:1890–8. 34431625 10.1111/os.13142 PMC8523760

[4] Rivera VR , Parks BG , Boucher HR . Longitudinal and axial stability of a cementless metaphyseal versus a fully porous coated cylindrical femoral stem. J Surg Orthop Adv. 2009;18:99–102. 19602338

[5] Song OK , Yong EC , Seo N , et al. Metal implants influence CT scan parameters leading to increased local radiation exposure: a proposal for correction techniques. PLoS One. 2019;14:e0221692. 31442288 10.1371/journal.pone.0221692 PMC6707604

[6] Binder EF , Brown M , Sinacore DR , Steger‐May K , Yarasheski KE , Schechtman KB . Effects of extended outpatient rehabilitation after hip fracture: a randomized controlled trial. Jama. 2004;292:837–46. 15315998 10.1001/jama.292.7.837

[7] Lee KJ , Um SH , Kim YH . Postoperative rehabilitation after hip fracture: a literature review. Hip Pelvis. 2020;32:125–31. 32953704 10.5371/hp.2020.32.3.125 PMC7476786

[8] Suetta C , Aagaard P , Rosted A , et al. Training‐induced changes in muscle CSA, muscle strength, EMG, and rate of force development in elderly subjects after long‐term unilateral disuse. J Appl Physiol. 1985;2004(97):1954–61. 10.1152/japplphysiol.01307.2003 15247162

[9] Weinstein RS , Lopez AM , Joseph BA , et al. Telemedicine, telehealth, and Mobile health applications that work: opportunities and barriers. Am J Med. 2014;127:183–7. 24384059 10.1016/j.amjmed.2013.09.032

[10] Maresca G , Maggio MG , De Luca R , et al. Tele‐neuro‐rehabilitation in Italy: state of the art and future perspectives. Front Neurol. 2020;11:563375. 33101176 10.3389/fneur.2020.563375 PMC7554582

[11] Aloyuni S , Alharbi R , Kashoo F , et al. Knowledge, attitude, and barriers to Telerehabilitation‐based physical therapy practice in Saudi Arabia. Healthcare. 2020;8:460. 33158298 10.3390/healthcare8040460 PMC7711516

[12] Jansen‐Kosterink S , In't Veld RH , Hermens H , Vollenbroek‐Hutten M . A telemedicine service as partial replacement of face‐to‐face physical rehabilitation: the relevance of use. Telemed E Health. 2015;21:808–13. 10.1089/tmj.2014.0173 26431260

[13] Galea MD . Telemedicine in rehabilitation. Phys Med Rehabil Clin. 2019;30:473–83. 10.1016/j.pmr.2018.12.002 30954160

[14] Zhang YY , Zhang YG , Li Z , Li SH , Xu WG . Effect of home‐based telerehabilitation on the postoperative rehabilitation outcome of hip fracture in the aging population. Orthop Surg. 2022;14:1768–77. 35819099 10.1111/os.13293 PMC9363742

[15] Knobe M , Siebert CH . Hip fractures in the elderly: osteosynthesis versus joint replacement. Orthopade. 2014;43:314–24. 24615534 10.1007/s00132-014-2265-7

[16] Liu Y , Chen X , Zhang P , Jiang B . Comparing total hip arthroplasty and hemiarthroplasty for the treatment of displaced femoral neck fracture in the active elderly over 75 years old: a systematic review and meta‐analysis of randomized control trials. J Orthop Surg Res. 2020;15:215. 32527294 10.1186/s13018-020-01725-3 PMC7291510

[17] Coulter C , Perriman DM , Neeman TM , Smith PN , Scarvell JM . Supervised or unsupervised rehabilitation after total hip replacement provides similar improvements for patients: a randomised controlled trial. Arch Phys Med Rehabil. 2017;98:2253–64. 28506775 10.1016/j.apmr.2017.03.032

[18] Harris WH . Traumatic arthritis of the hip after dislocation and acetabular fractures: treatment by mold arthroplasty. An end‐result study using a new method of result evaluation. J Bone Jt Surg. 1969;51:737–55. 5783851

[19] Mackintosh S . Functional independence measure. Ann Phys Rehabil Med. 2011;54:e237.

[20] Jegede RO . Psychometric attributes of the self‐rating anxiety scale. Psychol Rep. 1977;40:303–6. 840986 10.2466/pr0.1977.40.1.303

[21] Sciard D , Cattano D , Hussain M , Rosenstein A . Perioperative management of proximal hip fractures in the elderly: the surgeon and the anesthesiologist. Minerva Anestesiol. 2011;77:715–22. 21283071

[22] Saltvedt I , Prestmo A , Einarsen E , Johnsen LG , Helbostad JL , Sletvold O . Development and delivery of patient treatment in the Trondheim hip fracture trial. A new geriatric in‐hospital pathway for elderly patients with hip fracture. BMC Res Notes. 2012;5:355. 22800378 10.1186/1756-0500-5-355 PMC3463430

[23] Min K , Beom J , Kim BR , et al. Clinical practice guideline for postoperative rehabilitation in older patients with hip fractures. Ann Rehabil Med. 2021;45:225–59. 34233406 10.5535/arm.21110 PMC8273721

[24] Huber J , Lisiński P . Early results of supervised versus unsupervised rehabilitation of patients with cervical pain. Int J Artif Organs. 2019;42:695–703. 31177899 10.1177/0391398819853296

[25] Jørgensen PB , Bogh SB , Kierkegaard S , et al. The efficacy of early initiated, supervised, progressive resistance training compared to unsupervised, home‐based exercise after unicompartmental knee arthroplasty: a single‐blinded randomized controlled trial. Clin Rehabil. 2017;31:61–70. 27029938 10.1177/0269215516640035

[26] Ju JB , Zhang PX , Jiang BG . Risk factors for functional outcomes of the elderly with intertrochanteric fracture: a retrospective cohort study. Orthop Surg. 2019;11:643–52. 31456321 10.1111/os.12512 PMC6712441

[27] Latham NK , Harris BA , Bean JF , et al. Effect of a home‐based exercise program on functional recovery following rehabilitation after hip fracture: a randomized clinical trial. Jama. 2014;311:700–8. 24549550 10.1001/jama.2014.469 PMC4454368

[28] Fan XM , Bi ZG , Fu CJ , et al. Clinical study of psychological changes and post traumatic stress disorder in elderly patients with hip fracture. Zhonghua Wai Ke Za Zhi. 2020;58:209–12. 32187924 10.3760/cma.j.issn.0529-5815.2020.03.008

[29] Gambatesa M , D'Ambrosio A , D'Antini D , et al. Counseling, quality of life, and acute postoperative pain in elderly patients with hip fracture. J Multidiscip Healthcare. 2013;6:335–46. 10.2147/JMDH.S48240 PMC3785381 24082786

[30] Peeters CM , Visser E , Van de Ree CL , Gosens T , Den Oudsten BL , De Vries J . Quality of life after hip fracture in the elderly: a systematic literature review. Injury. 2016;47:1369–82. 27178770 10.1016/j.injury.2016.04.018

[31] Lin S , Xiao LD , Chamberlain D . A nurse‐led health coaching intervention for stroke survivors and their family caregivers in hospital to home transition care in Chongqing, China: a study protocol for a randomized controlled trial. Trials. 2020;21:240. 32131876 10.1186/s13063-020-4156-z PMC7057579

[32] Creasy KR , Lutz BJ , Young ME , Stacciarini JM . Clinical implications of family‐centered care in stroke rehabilitation. Rehabil Nurs. 2015;40:349–59. 25648522 10.1002/rnj.188 PMC4544639

[33] Australian and New Zealand Society for Geriatric Medicine . Position statement—dysphagia and aspiration in older people. Australas J Ageing. 2011;30:98–103. 21672120 10.1111/j.1741-6612.2011.00537.x

[34] Jiang Y , Luo Y , Li J , et al. Chronic kidney disease and risk of postoperative cardiovascular events in elderly patients receiving hip fracture surgery. Injury. 2022;53:596–602. 34974909 10.1016/j.injury.2021.12.032

[35] Cheung WH , Shen WY , Dai DL , et al. Evaluation of a multidisciplinary rehabilitation programme for elderly patients with hip fracture: a prospective cohort study. J Rehabil Med. 2018;50:285–91. 29260234 10.2340/16501977-2310

[36] Piga M , Cangemi I , Mathieu A , Cauli A . Telemedicine for patients with rheumatic diseases: systematic review and proposal for research agenda. Semin Arthritis Rheum. 2017;47:121–8. 28420491 10.1016/j.semarthrit.2017.03.014

